# Tongue Reconstruction with Buccinator Myomucosal Island Flaps: Technical Considerations, Oncologic Safety, Functional Outcomes and QoL Assessment—A Retrospective Observational Study

**DOI:** 10.3390/jpm13060879

**Published:** 2023-05-23

**Authors:** Olindo Massarelli, Luigi Angelo Vaira, Salvatore Crimi, Giovanni Salzano, Linda Latini, Alberto Bianchi, Paolo Gennaro, Giacomo De Riu

**Affiliations:** 1 Maxillofacial Surgery Operative Unit, Department of Mental Health and Sense Organs, Santa Maria Le Scotte, University Hospital of Siena, 53100 Siena, Italy; molindo74@gmail.com (O.M.); latilinda94@gmail.com (L.L.); paolo.gennaro@unisi.it (P.G.); 2 Maxillofacial Surgery Operative Unit, Department of Medicine, Surgery and Pharmacy, University of Sassari, 07100 Sassari, Italy; gderiu@uniss.it; 3 Biomedical Science Department, PhD School of Biomedical Science, University of Sassari, 07100 Sassari, Italy; 4 Maxillofacial Surgery Unit, University Hospital “Policlinico San Marco” of Catania, 95124 Catania, Italy; tore.crimi@gmail.com (S.C.); alberto.bianchi@unict.it (A.B.); 5 Maxillofacial Surgery Operative Unit, University Hospital of Naples “Federico II”, 80131 Naples, Italy; giovannisalzanomd@gmail.com

**Keywords:** tongue reconstruction, cheek myomucosal flap, FAMM, BAMMIF, facial artery myomucosal island flap, buccinator myomucosal flap, buccal artery myomucosal island flap, maxillofacial surgery, t-FAMMIF

## Abstract

The objective of this study was to investigate the effectiveness of buccinator myomucosal island flaps for tongue reconstruction following malignant tumor resections. A retrospective study was performed on 52 patients who underwent tongue reconstructions with buccinator myomucosal island flaps between 2012 and 2020. We reviewed the flap type and size, harvesting time, recipient- and donor-site complications, postoperative oncologic outcomes, functional recovery and QoL assessment. All of the flaps were transposed successfully without any total flap loss. Neither in the primary site nor in the neck were cancer relapses observed. An evaluation of the sensitivity revealed that 96.1% of patients experienced a recovery of touch, two-point and pain sensations. There were significant differences between the flap and the native mucosa in terms of the tactile (*p* < 0.001), pain (*p* < 0.001) and two-point (*p* < 0.001) thresholds. The average swallowing score recorded was 6.1 out of 7, with only minor complaints. The quality of life assessments demonstrated high scores across physical (24.5 out of 28), social (25.8 out of 28), emotional (20.3 out of 24) and functional (25 out of 28) aspects. The present study showed how buccinator myomucosal island flaps represent an effective and functional tongue reconstructive option, requiring a short operative time with a low rate of donor site morbidity, and with evidence of long-term oncologic safety and high quality of life.

## 1. Introduction

Reconstruction following the resection of malignant tongue tumors is a significant challenge in oral oncology [1,2,3]. The tongue is a crucial structure in the oral phase of swallowing, and is responsible for propelling food into the pharyngeal phase. It also plays a vital role in speech articulation, taste and protection of the upper airways [4,5]. Reconstructing the human tongue is a challenging endeavor due to its highly dynamic muscular structure, which cannot be precisely replicated using any existing technique. The primary objectives of reconstruction efforts should, therefore, focus on recreating the tongue’s integrity, volume and dynamic motion as faithfully as possible, while also restoring its sensitivity. This intricate process underscores the complexities involved in replicating such a unique and versatile organ. Numerous tongue reconstruction techniques are currently available. These methods range from direct closure [6,7], local flaps [8,9,10], regional flaps [11,12] to free flaps [13,14], each offering different benefits and challenges. While radial forearm (RF) and anterolateral thigh free flaps (ALT) are currently the gold standard for tongue reconstruction, providing satisfactory functional rehabilitation [15,16], buccinator myomucosal island flaps offer a promising alternative. Buccinator myomucosal island flaps are advantageous due to their minimization of donor-site morbidity and reduced surgery and hospitalization time. They provide a reconstructive option that offers the advantage of reconstruct the tongue with tissue that closely resembles the lost one in terms of texture, pliability, sensitivity and dynamic function. This similarity plays a crucial role in achieving successful functional outcomes [9,17,18,19,20,21,22,23,24].

The aim of this study was to evaluate the functional outcomes of buccinator myomucosal flaps for tongue reconstruction, and to establish their oncological safety.

## 2. Materials and Methods

**Figure 1:**
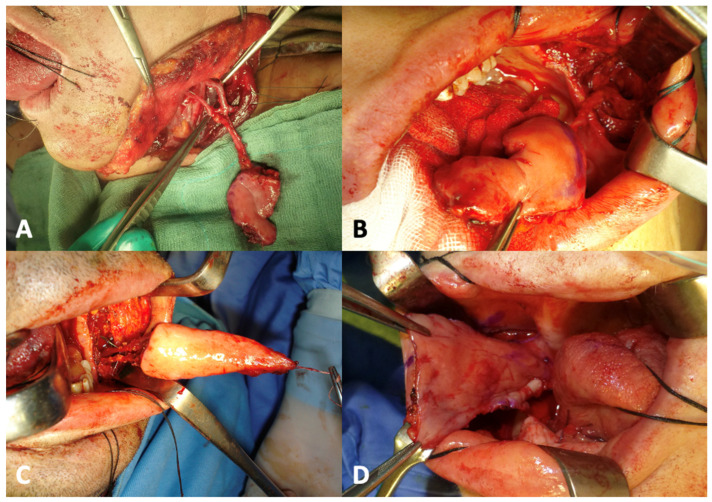
Types of buccinator myomucosal island flaps that can be harvested on the cheek. (**A**): tunnelized facial artery myomucosal island flap. (**B**): arterialized facial artery myomucosal island flap. (**C**): facial artery myomucosal island flap. (**D**): buccal artery myomucosal island flap.

A retrospective study was conducted in 52 patients with tongue oncological defects, who underwent reconstruction with buccinator myomucosal island flaps between February 2012 and July 2020 at the University of Sassari Maxillofacial Surgery Operative Unit. The flap harvesting was performed as described in previous reports [20] (Figure 1).

Patients were excluded from the study if they had a history of previous head and neck tumors, major surgery, or radiotherapy involving the oral cavity, or in case of incomplete data. Furthermore, individuals with potential conditions or a history of altered tongue sensitivity were not considered for the analysis. Similarly, patients who experienced a recurrence, or who had passed away within the first 12 months after surgery, were excluded from the analysis of functional data, but were considered in the analysis of oncological safety. The reason for this was because they were not able to undergo the 12-month postoperative evaluation.

All of the cases were evaluated by a multidisciplinary team that included a head and neck surgeon, a speech therapist and a psychologist. Following the hospital’s protocol, the patients who underwent oral cavity reconstruction were routinely subjected to a standardized evaluation of sensitivity recovery, swallowing function, quality of life (QoL) and donor site morbidity. This assessment took place 12 months after the surgery or upon completion of any adjuvant therapy, if performed [23,24]. The functional rehabilitation of patients with oral cavity tumors is one of the objectives established by the diagnostic–therapeutic pathway at the University Hospital of Sassari for patients with head and neck neoplasms. For this reason, periodic functional evaluation through standardized protocols holds a dedicated space in the clinical records of patients. These records were compiled during follow-up by the multidisciplinary team evaluating sensitivity recovery, complications, deglutition, speech, psychological well-being and quality of life.

The recovery of sensation was assessed in a quiet environment through various sensory domains: tactile sensitivity and pressure threshold, static and dynamic two-point discrimination, pain sensitivity, sharp/blunt discrimination and temperature sensitivity. The participants were blindfolded throughout all of the evaluations, in order to ensure unbiased results. Assessment tests for the recovery of sensitivity were conducted on both the surface of the flap and the healthy contralateral side, which served as a control site for comparison. This approach allowed for a thorough evaluation of the regained sensitivity by comparing the outcomes from the treated area with those of the unaffected, healthy side.

For the assessment of tactile sensitivity, a Semmes–Weinstein monofilament full set was used (Henry Schein, New York, NY, USA), with a pressure range varying from 0.0354 g/mm^2^ to 732.8 g/mm^2^. Following the test protocol, each nylon monofilament was pressed against the surface to be evaluated until it bent into a C-shape. The pressure was maintained for 1.5 **s**. The tactile threshold determination test always began with the thinnest monofilament, moving on to a thicker one in case the patient did not detect the pressure [25,26]. When the patient reported perceiving the touch of the monofilament, the corresponding pressure was recorded as the tactile sensitivity threshold.

The patient’s two-point discriminative sensitivity, both static and dynamic, was assessed using sterile staples with predetermined widths ranging from 1 to 30 mm. The staples were held with a needle holder, and gently placed (for static two-point sensitivity) or dragged (for dynamic two-point sensitivity) across the mucosal surface. The evaluation always began with the narrowest staple, asking the patients if they detected one or two stimuli. In case of an incorrect response, larger width staples were used until the patient could detect the stimuli as two and separate [19]. The thermal sensitivity was assessed using three cotton swabs soaked in water at 70 °C, cooled with ice to 3 °C, or at room temperature. The swabs were applied to the mucosal surface in random order, asking the patients which type of stimulus they perceived (warm, cold or neutral). The patient’s pain sensitivity was assessed with a prick test, using surgical forceps to pinch the test surface and asking the patients if they felt pain. The pain sensitivity threshold was assessed using the Semmes–Weinstein monofilament set, applied in the same manner as for the tactile sensitivity threshold assessment. Starting with the value determined for the latter, thicker monofilaments were used until the patient reported feeling pain in addition to pressure. If the patient did not feel pain, even with the thickest monofilament (732.8 g/mm^2^), this value was recorded as the pain sensitivity threshold [27]. The blunt/sharp discriminative sensitivity assessment was performed using a cotton swab and a dental probe. These tools were applied gently to the test surface in a random manner, asking the patients if they could distinguish the type of stimulus.

Swallowing was assessed according to the protocol described by Teichgraeber et al. [28]. The patient was administered foods of three different consistencies: liquid (still water), semiliquid (yogurt lactose-free) and gelatinous (water-based gel). For each consistency, a quantity equivalent to a spoonful was provided. Following this, the patient was asked to perform a swallowing action. The ability to swallow effectively was evaluated by assessing the amount of food residue left on the palate following the swallowing action. The effectiveness and quality of swallowing were subsequently classified on a scale of 1 to 7. A score of 1 indicates severe complaints and an inability to swallow, while a score of 7 signifies no complaints and an effective swallowing process. The overall score was determined by the mean of the scores for the three foods administered. This procedure, however, deviated slightly from the protocol established by Teichgraeber et al., as no solid foods were used. The rationale behind this exclusion was the recognition that the swallowing of solid foods is significantly influenced by a patient’s mastication capabilities [28]. Therefore, solid foods were omitted from this evaluation to focus solely on the act of swallowing and its related mechanisms, without the potential confounding influence of mastication.

The quality of life levels were studied using the Functional Assessment of Cancer Therapy—Head and Neck Version questionnaire [29]. Finally, morbidity at the cheek donor site was assessed as described by Ferrari et al. [30], by a panel of three researchers and the patients themselves. Five parameters were evaluated: mouth opening, mucosal lining symmetry of the cheek, cheek vestibule depth, buccal commissure symmetry, and overall aesthetic quality. Each parameter was given a score of 0 to 3, with the scores summed to obtain an overall assessment.

The functional outcome data were extracted from patients’ clinical records, along with information on the type, location and dimension of the tumor, the type of myomucosal flap used, the duration of the surgical procedure, and any intraoperative, perioperative and postoperative complications. Additionally, records were examined for any instances of cancer recurrence.

The data were collected and analyzed using the Statistical Package for the Social Sciences (IBM SPSS 24.0.0, SPSS Inc, Chicago, IL, USA). Descriptive statistics are provided in the form of the mean ± standard deviation (SD) or the median [interquartile range]. Differences in the tactile and pain sensitivity thresholds between the reconstructive flap and healthy contralateral side were analyzed using the Wilcoxon signed-rank test for paired data. Differences in the swallowing scores between groups selected according to the T stage were analyzed by means of a one-way ANOVA with the Games–Howell post hoc test. Spearman’s correlation analysis was performed to assess the relationship between the swallowing scores and flap size. The statistical significance level was set at ***p*** ≤ 0.05 with a 95% confidence interval.

This study was conducted in accordance with the Helsinki Declaration of 1973, as revised in 1983. As a retrospective study, it did not require institutional review board approval.

## 3. Results

Out of 63 patients who underwent tongue reconstruction using buccinator myomucosal flaps between January 2012 and January 2020, two passed away due to comorbidities and one to pulmonary metastasis in the first 12 months after surgery. Eight patients did not undergo the 12-month functional evaluation, so they were excluded. The study included the remaining 52 patients (37 males, 15 females, mean age 58.7 years), analyzing demographic data, tumor pathology, reconstructive flap type and size, harvesting times, postoperative complications and adjuvant radiotherapy (Table 1).

The patients were classified as T1 in 5 cases, T2 in 32 cases, T3 in 12 cases and T4 in 3 cases. The tumor resection was combined with lymph node neck dissection based on oncological guidelines. In 42 patients with cN0 staging, the facial artery and vein were preserved during the dissection of the neck, allowing the buccinator myomucosal island flap to be harvested on the facial vessels. Six patients with cN0 disease were upstaged to pN2b or pN3b after the histological evaluation. In eight cases, staged as N1 and N2, the flaps were based on the buccal artery. One cN2b case and one case with a prior facial artery traumatic section used flaps that were based on the contralateral facial artery, due to its sufficient pedicle length. The average flap harvesting time was 49.4 min.

No complete flap loss or major complications were reported. Three minor complications were detected: one case of venous stasis that resolved completely and spontaneously, and two cases of marginal necrosis with minor dehiscence of the surgical wound. Thirteen patients (25%) underwent adjuvant radiotherapy. During the 12-month follow-up, no recurrences were detected in the series. Two patients died of causes that were unrelated to tongue cancer, while one patient, affected by advanced adenoid cystic carcinoma, died of lung metastases. The other eight patients lost during follow-up had been referred to our disease treatment center from other regions, and continued to be followed up at the referral center in their home territory. During the subsequent follow-up (median follow-up 53 months, range 124–126 months), one patient developed a recurrence on the tongue, while lymph node recurrence occurred in three cases. All of these patients had an initial N+ staging whereby the facial vessels were sacrificed during the neck dissection, and the flap was pedicled over the buccal vessels. No case of lymph node recurrence was detected in the cN0 patients, not even in those up-staged to N+ after the histopathological examination.

At the sensitivity evaluation, 50 patients (96.1%) demonstrated a recovery of tactile, static and dynamic two-point discriminative and pain sensitivity. Blunt/sharp discriminative sensitivity was found to be absent in seven cases, while eight patients were unable to distinguish between hot and cold stimuli. Significant differences were observed between the reconstructed flap and the healthy contralateral side for the tactile (*p* < 0.001) and pain (*p* < 0.001) sensitivity thresholds, as well as for static (*p* < 0.001) and dynamic (*p* < 0.001) two-point discrimination (Table 2).

**Figure 2:**
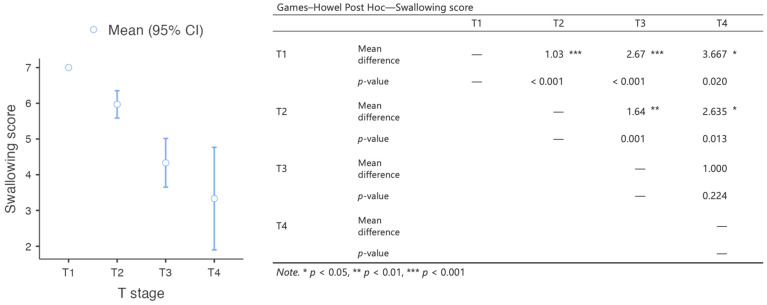
Results of the analysis of the differences in swallowing scores between groups of patients with different T staging.

Most patients experienced minimal swallowing complaints, and could swallow without difficulty. The median deglutition score was 6 [IQR 5–7]. The differences in the swallowing scores between groups selected on the basis of the T stage were statistically significant, with an inversely proportional correlation between tumor size and swallowing quality (Figure 2). A moderate and significant inverse relationship was detected between the swallowing scores and flap size (r_s_ = −0.458, *p* < 0.001).

The quality of life assessment indicated a satisfactory recovery of physical, social, emotional and functional parameters (Table 3).

In all of the cases, the donor site was filled with the buccal fat pad from the cheek to promote healing. The donor site morbidity outcome was generally acceptable, resulting in a low overall impact on the patients. (Table 4). Five patients required a secondary Z-plasty due to cheek scar retraction.

## 4. Discussion

The tongue is a vital organ for the proper execution of essential functions in an individual. It plays a primary role in the articulation of language, and in the correct pronunciation of phonemes. Moreover, it is crucial for the proper distribution of food within the mouth during mastication, and for pushing the bolus into the hypopharynx during swallowing. By channeling food and saliva into the digestive tract, it also has a fundamental function in protecting the airways. Furthermore, the tongue is the seat of taste, allowing individuals to appreciate the food they consume, transforming eating from a mere necessity for survival into a pleasurable experience. All of these functions are ensured by the tongue’s high specialization. The integrity of its intrinsic and extrinsic musculature and the sensitivity of its mucosa are fundamental for preserving its function [14]. Consequently, tongue defects resulting from ablative surgery or trauma, even if small, can have devastating effects on patients’ quality of life.

**Figure 3:**
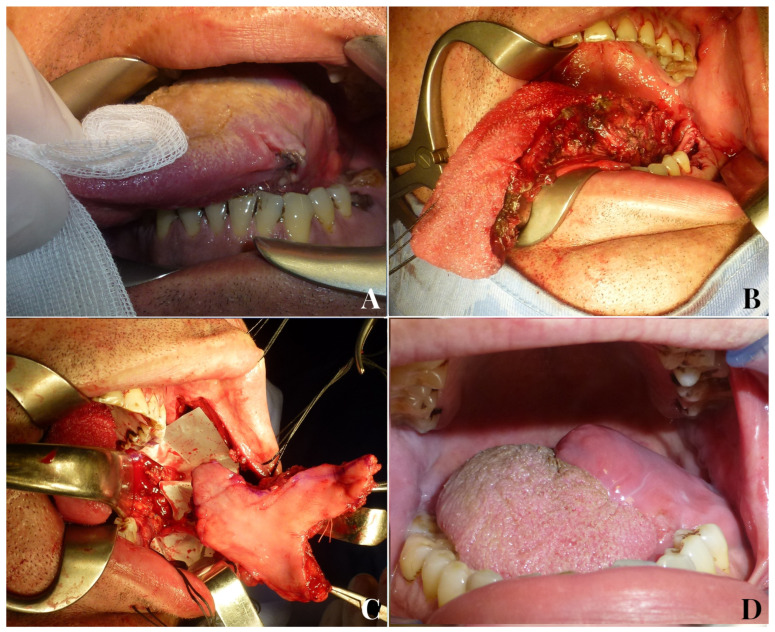
A 51-year-old male patient with endophytic squamocellular carcinoma of the left tongue (**A**). Left hemiglossectomy defect following tumor ablation (**B**). A facial artery myomucosal flap (FAMMIF) was harvested from the left cheek to reconstruct the tongue defect (**C**). Follow-up at 14 months after surgery (**D**).

The goals of an ideal tongue reconstruction should be to achieve watertight closure of the defect, preventing saliva leakage into the neck and recreating a stable separation between anatomical structures; to avoid limitations in tongue mobility due to scar retractions; to facilitate tongue movement by reconstructing the lost muscular portion; and to restore mucosal sensitivity, with the ultimate aim of reestablishing tongue function as close to the original as possible [31] (Figure 3). Unfortunately, there are currently no reconstructive techniques that can accurately replicate the complex myomucosal structure of the tongue.

Several reconstructive techniques have been proposed, including primary closure [6,7] and the use of local, [8,9,10] regional [9] and distant free flaps [13,14]

In the cases of patients with small partial glossectomies, affecting at most a quarter of the tongue, direct suturing of the defect or the use of split-thickness skin grafts can yield excellent functional results. However, this reconstructive approach cannot be used for larger defects, as it does not allow for the restoration of lost tongue volume, which would be too small to reestablish normal function [6,7]. For larger defects, or if the floor of the mouth is also involved, flap reconstruction is required to restore adequate tongue bulk, prevent ankyloglossia and improve functional recovery.

The pectoralis major musculocutaneous flap was the primary means of head and neck reconstruction before the advent of the free flaps [12]. The main disadvantages of this flap include its excessive bulk and poor pliability of the skin paddle, its limitations in pedicle length and compromised tongue elevation by the muscle’s downward traction.

Actually, the first choice for tongue reconstruction is represented by free flaps as radial forearm flap (RF) or anterolateral thigh flap (ALT). The RF presents the following advantages: it has a consistent anatomy; it can be harvested simultaneously with oral ablation; it has reinnervation potential; it is thin and pliable; it has a long pedicle and caliber vessels that are large enough to allow easier microvascular anastomosis [31]. However, the RF can leave an evident scar on the wrist, and sometimes can lead to hand numbness and weakness. Furthermore, the RF typically provides less tissue than other free-flap sources, resulting in insufficient bulk for the reconstruction of sub-total or total glossectomies. The ALT flap is bulkier and produces a lower donor site morbidity. For this reason, it is generally preferred to the RF for the reconstruction of larger tongue defects.

However, with these techniques, the transplanted tissues do not provide sensitivity, mobility, volume or texture that are similar to the native mucosa. For this reason, several authors proposed sensory reinnervation of the RT and ALT free flaps to achieve sensitivity recovery that is crucial to improve oral function [32]. Therefore, non-reinnervated free flaps can recover sensitivity in 20–70% of the cases, while reinnervated ones recover in 60–100% of the cases, significantly improving speech and swallow [32,33,34,35,36,37]. Finally, although these flaps provide an excellent reconstruction for large defects, they result in excessive bulk for medium-sized defects.

**Figure 4:**
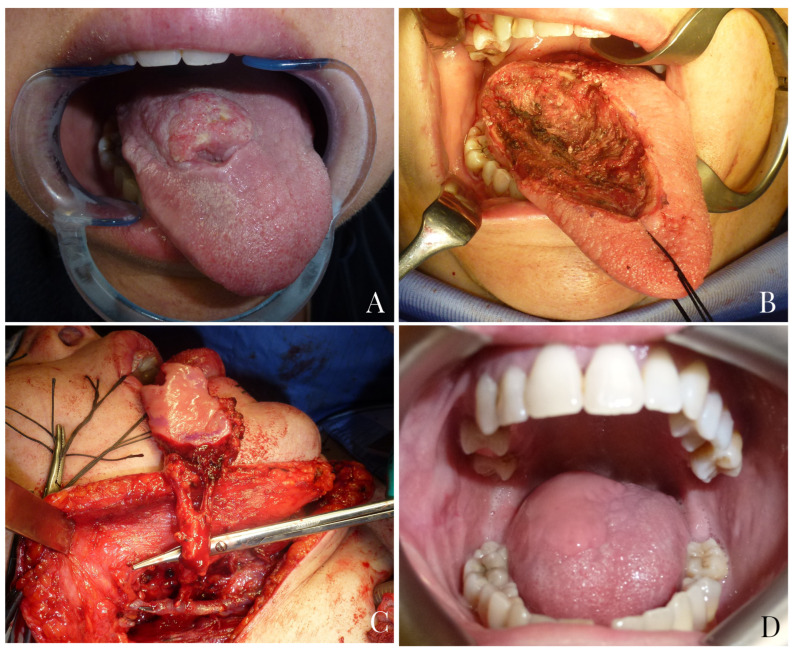
A 45-year-old female patient with squamocellular carcinoma of the right tongue (**A**). Right tongue defect following tumor resection (**B**). Tunnelized facial artery myomucosal flap (t-FAMMIF) was harvested from the right cheek to reconstruct the tongue defect (**C**). Follow-up at 25 months after surgery (**D**).

In 2013, Massarelli et al. [17] reported their experience on oral cavity reconstruction with buccinator myomucosal island flaps. Buccinator myomucosal flaps are based on the anastomotic network between the facial and buccal arteries. They allow for the transfer of tissue to the recipient site that is as similar as possible to the lost tissue in terms of texture, thinness, mobility and vascularization. Moreover, the flap’s mucosa helps maintain mucosal secretions and sensitivity, which are essential for the rehabilitation of tongue functions such as speech articulation and swallowing. Thanks to the tunneling technique, it is possible to significantly expand the rotation arc of the flap and, most importantly, to avoid a second surgical stage for pedicle division [9,20] (Figure 4).

Buccinator myomucosal flaps, compared to free flaps, can be harvested more quickly, on the same surgical field, and without requiring a second surgical team. In this way, operative times and costs are reduced, additional surgical sites are not needed, which results in less morbidity and no visible skin scar [23].

In patients classified as cN+ and in those cases in which the facial artery was ligated or accidentally cut, a buccinator myomucosal flap may be harvested on the buccal vessels (BAMMIF) [20]. The rotation pivot of this flap, centered on the pterigo-mandibular raphe, make it effective for the reconstruction of the posterior half of the mobile tongue. It must be clear that to ensure oncological radicality, myomucosal flaps based on facial vessels are not indicated in cases of clinically or radiologically evident lymph node metastases, where radical neck dissection, necessitating the sacrifice of the facial artery and vein, must therefore be performed. The oncological safety of preserving facial vessels in the case of selective dissection has already been reported for both submental [38,39,40] and myomucosal [41] flaps. Similarly, in this series, we did not observe a higher rate of lymph node recurrence in cN0 patients where the facial vessels were preserved, even in the cases that were up-staged to N+ after histological examination.

To the best of our knowledge, this is the first study to evaluate tongue functional recovery with buccinator myomucosal island flaps in a so large a case series. Our results showed excellent functional rehabilitation. The sensitivity assessment showed that touch, two-point and pain sensations were recovered in 96.1% of the patients; only seven patients were not able to discriminate between sharp and blunt stimuli, while only eight patients did not report thermal sensitivity (Table 2). These brilliant results, even better than those reported for fasciocutaneous reinnervated free flap reconstructions [31,32,33,34,35,36,37], may be related, in our opinion, to the low fibrotic retraction of the buccinator muscle which favors nerve sprouting from the surrounding tissues [19,23,24,25]. Moreover, most of the patients presented minimal deglutition complaints, and were able to swallow boluses without any difficulty, and had a satisfactory quality of life level (Table 3).

In all of the cases, the donor site was repaired with a buccal fat pad harvested from the cheek, leading to low donor site morbidity (Table 4). Trismus may occur due to the donor site scar, but this can be avoided with postoperative massages. In five patients of our series, scar retraction on the cheek required a secondary Z-plasty.

The limitations of the present study are mainly due to its retrospective nature. Without preoperative evaluations, it would not have been possible to fully understand how the surgery influenced the outcomes observed during the follow-up assessments. However, at least regarding the sensitivity assessment, the healthy contralateral side provided a reliable comparison. Additionally, this study did not include a control group of patients who underwent alternative surgical techniques; hence, future research comparing the results of myomucosal flap reconstructions with those of fasciocutaneous free flaps will be necessary. Despite these limitations, this study has the significant strength of being the first to analyze such a large cohort of patients with the same type of post-resection defect and utilizing a comprehensive assessment protocol.

## 5. Conclusions

**Figure 5:**
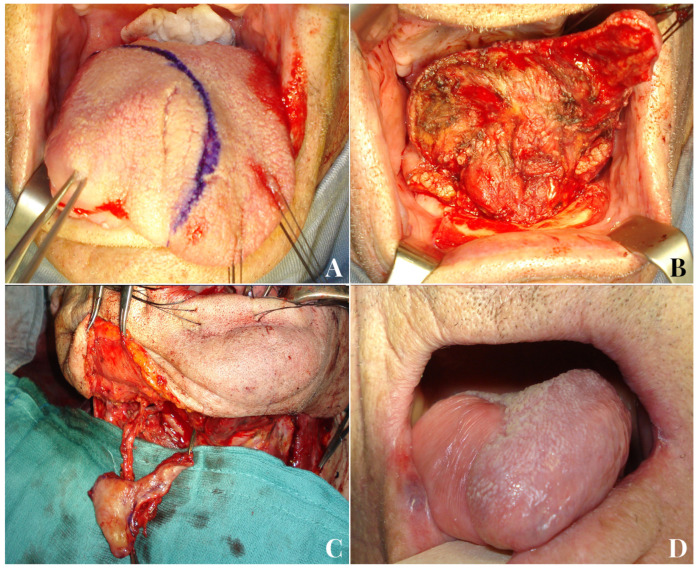
An 81-year-old male patient with squamocellular carcinoma of the right tongue (**A**). Right hemiglossectomy defect following tumor ablation (**B**). A tunnelized facial artery myomucosal flap (t-FAMMIF) was harvested from the right cheek to reconstruct the tongue defect (**C**). Follow-up 12 months after the end of radiotherapy (**D**).

Concerning tongue reconstruction, in our opinion, buccinator myomucosal flaps, especially declined in the form of t-FAMMIF, represent the first reconstructive option for medium defects up to hemiglossectomy (Figure 5).

Free flaps remain a suitable choice in the case of a pull-through hemiglossectomy, total glossectomy or evidence of neck metastases. Compared to free flaps, they do not require two surgical teams, they cause less donor morbidity due to no evident scarring and considerably reduce operating times as well as post-surgical complications.

The preservation of facial vessels does not seem to undermine the oncological safety of neck lymph node dissection in cN0 patients. However, the indication for the execution of a myomucosal flap based on facial vessels is strictly limited to patients who do not show suspected metastases in preoperative examinations.

Based on the results of this study, buccinator myomucosal flaps have demonstrated significant recovery of sensitivity in the vast majority of cases, allowing for like-with-like reconstruction of the tongue. This facilitates a more straightforward and quicker recovery of swallowing function, which was confirmed by the evaluation conducted in this study. Ultimately, functional recovery is fundamental to restoring satisfactory quality of life to the patient.

## Figures and Tables

**Table 1:** Patients’ characteristics.

	N (%)
Age	
<55	16 (30.8%)
56–74	29 (55.8%)
>75	7 (13.4%)
Gender	
Male	37 (71.2%)
Female	15 (28.8%)
Etiology	
Squamocellular carcinoma	51 (98.1%)
Adenoid-cystic carcinoma	1 (1.9%)
T Stage	
T1	5 (9.6%)
T2	32 (61.5%)
T3	12 (23.1%)
T4	3 (5.8%)
N stage	
N0	36 (69.2%)
N1	1 (1.9%)
N2	11 (21.1%)
N3	4 (9.6%)
Radiotherapy	
Yes	13 (25%)
No	39 (75%)
Type of reconstruction ^1^	
FAMMIF	14 (26.9%)
t-FAMMIF	27 (51.9%)
a-FAMMIF	3 (5.8%)
BAMMIF	8 (15.4%)
Ipsilateral myomucosal flap	50 (96.2%)
Contralateral myomucosal flap	2 (3.8%)
Mean flap size (cm)	6.2 × 5.5
Mean harvesting time (minutes)	49.4 min
Type of neck dissection	
Unilateral	
Selective	28 (53.9%)
Modified radical	4 (7.7%)
Bilateral	
Selective + selective	18 (34.6%)
Selective + modified radical	2 (3.8%)
Modified radical + modified radical	0 (0%)

^1^ FAMMIF: Facial artery myomucosal island flap; t-FAMMIF: tunnelized facial artery myomucosal island flap; a-FAMMIF: arterialized facial artery myomucosal island flap; BAMMIF: buccal artery myomucosal island flap [20].

**Table 2:** Results of sensitivity assessment.

Soft Touch	Tactile Threshold (g/mm^2^)		Two-Point Discrimination Static/Dynamic (mm)		Prick Test	Pain Threshold (g/mm^2^)		Sharp/Smooth Discrimination	Hot/Cold Discrimination
	Flap	Contralateral/ Native Mucosa	Flap	Contralateral/ Native Mucosa		Flap	Contralateral/ Native Mucosa		
96.1%	0.664 ± 0.435	0.358 ± 0.008	13.7 ± 3.8/10.4 ± 4.1	6.1 ± 1.5/3.4 ± 1.2	96.1%	647.62 ± 176.39	188.58 ± 181.39	45 86.5%	44 84.6%
Statistical Analysis	Wilcoxon Test Z = −4.862 *p* < 0.001		Wilcoxon Test Static: Z = −6.282 *p* = 0.000 Dynamic: Z = −6.298 *p* < 0.001			Wilcoxon Test Z = −5.960 *p* < 0.001			

**Table 3:** Results of quality of life assessment through the Functional Assessment of Cancer Therapy—Head and Neck Scale (FACT-H&N) [29].

Quality of Life	Physical Well-Being (0–28)	Social/Family Well-Being (0–28)	Emotional Well-Being (0–24)	Functional Well-Being (0–28)	H&N Cancer Sub Scale (0–40)
Mean ± SD	24.5 ± 3.3	25.8 ± 2	20.3 ± 1.8	25 ± 2.6	37.25 ± 2.2

**Table 4:** Donor site morbidity assessment [30].

	Mouth Opening (1–9)	Commissure Symmetry (1–9)	Inner Vestibule (1–9)	Cheek Lining (1–9)	Esthetics (1–9)
Mean ± SD	7.7 ± 1.2	8.1 ± 0.8	7.8 ± 1	8.2 ± 0.7	8.2 ± 0.8

## Data Availability

The raw data included in this study are available from the corresponding author on request.
